# Conditioned Pain Modulation Effectiveness: An Experimental Study Comparing Test Paradigms and Analyzing Potential Predictors in a Healthy Population

**DOI:** 10.3390/brainsci10090599

**Published:** 2020-08-30

**Authors:** María del Rocío Ibancos-Losada, María C. Osuna-Pérez, María Yolanda Castellote-Caballero, Ángeles Díaz-Fernández

**Affiliations:** 1 Physiotherapy Clinic Center of Bartolomé Puerta, Jaén-23006, Spain; mril0001@red.ujaen.es; 2 Department of Health Sciences, University of Jaén, Jaén-23071, Spain; mycastel@ujaen.es (M.Y.C.-C.); andiaz@ujaen.es (Á.D.-F.)

**Keywords:** conditioned pain modulation (CPM), ischemic pain, cold pain, conditioning stimulus, stimulus parameter

## Abstract

Conditioned pain modulation (CPM) is an endogenous pain inhibition phenomenon that can be summarized simply as one type of pain being able to inhibit another, which must be in a remote area in relation to the first pain. We aimed to compare the effectiveness of four CPM test paradigms as well as the association of the CPM effect with potential predictors in 72 healthy volunteers. Pressure pain from an algometer was used as the test stimulus, and pain provoked by cold water or ischemic pressure was used as the conditioning stimulus, applied either sequentially or in parallel. No significant differences were found between the test paradigms, although the cold-parallel test showed the most significant effect size (ηP^2^ = 0.614). No association was found between the CPM effect and sociodemographic variables (age or sex), nor anxiety, depression, catastrophizing, previous history of pain or self-perceived pain tolerance. Nevertheless, a strong association was found between the CPM effect and individual affinity for the stimulus in participants who underwent the cold water test paradigm; this explained around 45% of the total CPM effect when the paradigm (cold water) coincided with personal affinity for the stimulus (“I prefer cold to heat”, “cold is not unpleasant”).

## 1. Introduction

A growing concept in the field of pain modulation is conditioned pain modulation (CPM). CPM involves an endogenous descending pain inhibitory pathway [1]. CPM is correlated with the phenomenon known as diffuse noxious inhibitory control (DNIC), which was studied in rats by Le Bars [2]. For this reason, in 2010, experts recommended calling it CPM when this concept was extrapolated and applied to humans [3].

The mechanism of action can be summarized as “pain inhibits pain” [4], that is, a nociceptive stimulus applied in one part of the body can inhibit pain in another part of the body when activating this phenomenon [5]. The methodological sequence to activate this process is as follows: (a) apply a first nociceptive stimulus (test stimulus, TS), (b) apply a second nociceptive stimulus (conditioning stimulus, CS), and (c) repeat the TS to check if the CS has activated the phenomenon and provoked an analgesic effect. Traditionally a single TS is applied, but some experts recommend testing with two test stimuli as a way of investigating the test stimuli, not in the context of requiring two test stimuli to elicit a CPM effect, given that lack of consensus regarding a ‘gold standard’ paradigm [6].

Investigations have demonstrated that descending influences on spinal nociceptive processing involve the periaqueductal gray, rostral ventromedial medulla and subnucleus reticularis dorsalis, leading to the description of this descending pain modulation pathway as a spino-bulbo-spinal loop [7]. However, some findings indicate that the CPM response does not exclusively involve a spino-bulbo-spinal pathway, and it seems that physiological and psychological/cognitive pathways also interact with this phenomenon [8]. Other studies have found an interaction between the CPM phenomenon and the cardiovascular system too [9].

The importance of this phenomenon lies in the fact that it could be used as a method to assess our body’s ability to inhibit pain [10]. It has been observed that this response occurs in healthy people, but in populations with chronic pain (fibromyalgia, migraine or irritable bowel syndrome) it could be decreased [11].

The CPM effect could be influenced by certain factors such as sex [12], age [12], alcoholism [13], sleep [13], socioeconomic status [12], education level [14], exercise [12], hormonal contraception [15], and ovulatory phase [12]. It is also influenced by psychological factors such as anxiety level, depression, and catastrophism, where it has been found that at a higher level of these variables, there is a lower CPM response [16].

Other variables investigated in the field of pain (although not frequently studied in the CPM phenomenon) are optimism, expectations and experience before treatment, which could influence pain modulation [17,18,19].

For this reason, the CPM effect has been investigated in clinical practice in various settings, for example, as a prognostic factor for postsurgical pain (populations with a lower CPM effect may present with more postoperative pain) [20] or as a method to determine the efficacy of pharmacological, psychological and/or physiotherapeutic interventions [10,21].

The main challenge when studying the CPM effect lies in the existence of different test paradigms in the scientific literature, which hampers the consistency and extrapolation of results. Furthermore, various questions remain unanswered, such as:

### 1.1. Which Nociceptive Stimulus, or Modality, Should Be Used? 

A wide variety of stimuli can be found in quantitative sensory tests (QSTs): thermal stimuli (heat by contact, cold water), ischemic (compression with a cuff), pressure (algometry), mechanical (Von Frey filaments), or electrical [10].

One of the most studied stimulus is a pressure pain threshold as the TS, which shows good intrasession and intersession reliability [22], and cold water immersion as the CS with excellent reliability [23].

### 1.2. At What Intensity Should the Stimuli Be Applied? 

It is common to apply a noxious stimulus between 40–60 on a 0 to 100 scale [24] or until pain threshold [21] to TS, but for CS it is accepted that it needs to be painful, with some conflicting results on the influence of its intensity on the CPM efficiency. Some studies have hypothesized that even when the intensity is increased, it will not produce a greater effect because it is a saturable phenomenon [25]. The pain rating of the CS increases because it is a static measure; still, CPM magnitude does not demonstrate such a pattern of significant increase because it represents a dynamic measure) [25].

### 1.3. Where Should the Stimuli Be Applied? 

In principle, this does not matter, as long as they are not applied to homotopic ipsilateral zones because it is hypothesized that this may not evoke a CPM effect [26].

### 1.4. For How Long Should the Stimuli Be Applied? 

No specific consensus exists in terms of duration. However, in general, it is recommended to allow sufficient time for the individual to reach the intensity that is considered a painful (but tolerable) stimulus and should activate the CPM pathway [27].

### 1.5. When Should the Stimuli Be Applied (Sequential or Parallel Paradigm)? 

Both methods are used, but the parallel process has been demonstrated to produce a greater activation of the phenomenon. However, some studies attribute this better effectiveness to the “distraction” phenomenon [24], so some experts prefer to use the sequential method to avoid this distraction [6].

### 1.6. How Should the CPM Effect Be Calculated?

To calculate CPM effect, one can compare the pressure pain or pressure pain threshold before and during/after application of the conditioning stimulus. However, one can also compare the numerical rating scale of a test stimulus applied at a predefined intensity before and during/after application of the conditioning stimulus [6]. The difference between the parameters obtained along the pain modulation test paradigm can be presented as absolute values or percent change always indicating if the results denote pain inhibition or pain facilitation [6].

Considering the above, and following a review of the scientific literature, four test paradigms were developed based on the best evidence and recommendations, with the primary objective being to determine if these test paradigms produced the CPM response and to compare the effectiveness of each in terms of effect size. The secondary objective was to analyze the possible influence of potential predictors that may be associated with the CPM effect.

## 2. Materials and Methods

### 2.1. Participants

A total of 72 healthy volunteers from Jaén province participated (45 men and 27 women), with a mean age of 33.19 ± 12.70 years. Recruitment was carried out via adverts on different social networks. Enrolment was conducted during September 2019. The selection method was based on convenience sampling. To be able to participate, volunteers needed to meet the following inclusion criteria: (1) be healthy, (2) be an adult (>18 years), and (3) have the capacity to communicate and understand the instructions. Exclusion criteria were the ingestion of analgesic medication in the 24 h before undergoing the test, taking regular medication, being pregnant and not having slept well the night before (i.e., for more than 6 h in a row).

This study was designed following the principles outlined in the Declaration of Helsinki and was approved by the Ethics Committee of Jaén University (ethic approval code: JUL.19/1.TFM). Participants were provided with detailed information regarding the project. Next, written informed consent was obtained from each participant, indicating their voluntary acceptance of participation in the study. Participants were randomly distributed into four groups. The allocation was balanced in terms of the number of participants (18 per group). The distribution of participants was decided using a computer random number generator. Data analysts were blinded to group assignments.

### 2.2. Procedures

All test paradigms involved the following procedures:

- Application of the “familiarization test”: an initial test aimed at familiarizing the participant with the pressure of the algometer, allowing the participant to be able to better discriminate and evaluate sensations during the study. In this test, the participant remains seated in a chair. The investigator (located behind the participant) applies a pressure progressively at three different points [25,28] in the neck region in the same order (two points at the superior fibers of the trapezius and one point on the scalene muscle) with a digital algometer (FPIX; Wagner Instruments, Greenwich, CT, USA) on the non-dominant side. The application of pressure to the three locations was predetermined and always in the same order. For each of the points, the participant should indicate when the pressure is perceived as painful with 6/10 intensity on the Numerical Rating Scale (NRS) [29]. At that instant, the investigator should stop applying pressure and record the pressure reading on the algometer (kg/cm^2^);
- Application of the test stimulus (TS) (20 min after): The same procedure as above was conducted but, this time, on the dominant side. The mean of the three recorded values was calculated, and this mean value was considered as the preconditioning stimulus (Pre-CS);
- Application of the conditioning stimulus (CS) and repetition of the test stimulus: The conditioning stimulus was applied immediately after the application of the TS. In this study, two possible noxious stimuli were evaluated (pain from cold water and pain due to ischemia), with two different methods of application (sequential and parallel). Four groups or test paradigms were obtained for the possible combinations.

### 2.3. Ischemic-Sequential Group

The CS was ischemic pain. Ischemia was caused by a blood pressure cuff, placed at the ankle of the non-dominant leg, and inflated to a pressure of 250–260 mmHg [21]. The participant, who was seated with their leg raised, had to extend and flex their ankle repeatedly to produce ischemia for one minute [21]. Following this minute, the participant was asked to assess the sensation of pain produced by the ischemia. If the pain sensation reached 6/10 on the NRS, this part of the test was ended; if not, they tried to continue until this score on the NRS was reached [21]. Once 6/10 was reached on the NRS, the cuff was deflated, the participant lowered their leg, and, in a continuous sequential manner, the TS was applied again. The participant was again asked to report when the algometer pressure produced a sensation of pain at 6/10 on the NRS for each of the three points. The mean of the three values obtained was calculated, and the mean value was considered as the post-conditioning stimulus (Post-CS) value.

### 2.4. Ischemic-Parallel Group

The participants in this group underwent the same procedure as the aforementioned group, but once 6/10 on the NRS was reached with ischemic pain induced by the inflated blood pressure cuff, the leg position was maintained, and the TS applied concurrently. From this point, they were requested to state when the pressure of the algometer produced a 6/10 on the NRS, while still receiving the CS following the parallel method.

### 2.5. Cold-Sequential Group

The CS for participants in this group was cold water. The participant remained seated and, on receiving the signal, were requested to submerge their non-dominant foot (3 cm above the lateral malleolus) [30] in a container of cold water (8–10 °C) [22]. The temperature was controlled by a water thermometer and the water maintained within this range by the container’s cooling system. After 30 s, participants were asked to assess the sensation of pain produced by the cold water (for safety, they did not exceed two minutes in the cold water test paradigm). If it were scored as 6/10 on the NRS, they would remove their foot from the water; if not, the foot was maintained submerged until 6/10 was reached [21]. Once this score was reached and the foot removed from the water, the TS was applied in a consecutive manner. Again, the participants were asked to indicate when the pressure of the algometer produced 6/10 pain on the NRS for each of the three points on the neck. As in all the test paradigms, the mean of the three values was recorded, and this mean value was considered as the post-conditioning stimulus (Post-CS) value.

### 2.6. Cold-Parallel Group

The same test paradigm was followed as for the cold-sequential group; however, when the participants approached 6/10 on the NRS, the TS was simultaneously applied, in such a way that while the foot was submerged, the participant had to indicate when the algometer pressure produced 6/10 on the NRS for each of the three points on the neck. The corresponding Post-CS value was again calculated.

**Figure 1:**
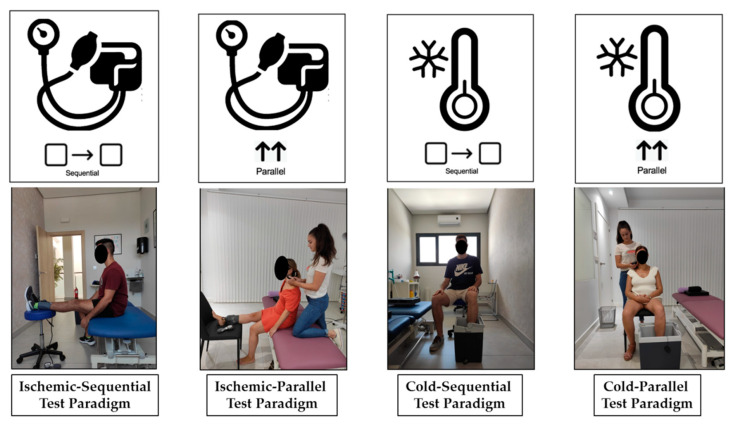
Images of the experimental setup (conditioned pain modulation (CPM) test paradigms).

Figure 1 shows summary images of the procedures.

Each subject and each test paradigm was identified with a random number, allowing the investigator to be blinded in the analysis of the data.

### 2.7. Primary Outcome Measure and Secondary Outcome Measures

#### 2.7.1. Principal Variable (CPM Effect)

The difference was calculated between the mean values obtained on application of the conditioning stimulus (Post-CS) and the mean value for pressure pain before the application of the conditioning stimulus (Pre-CS) [30]. Hence, a positive CPM effect indicated a reduction in pain (inhibition) or an increase in the pain tolerance, and therefore, that the CPM phenomenon had been activated. In contrast, a negative result indicated pain facilitation and that the CPM phenomenon had not been activated. The difference between the parameters obtained along the pain modulation test paradigm was presented as absolute values, indicating if the result denoted pain inhibition or pain facilitation [6].

#### 2.7.2. Other Variables

Sociodemographic, anthropometric, and healthy habit variables: For each participant, data were collected on sex, age (in years), and body mass index (calculated from data on weight and height using the following formula: Body Mass Index (BMI) = weight (kg)/height (m^2^)). Data were also collected on education level (compulsory secondary education, professional formation/degree or postgraduate), socioeconomic level (low or medium), physical activity level (in hours/week: <1 h, 1–3 h, >3 h per week), if they had any allergies, smoking and which was their dominant side (right- or left-handed). The variables menstrual cycle (“Do you have your period now?”: yes/no) and hormonal contraception (yes/no) were also taken into account.

#### 2.7.3. Anxiety Level 

This was measured with the Spanish version of Spielberger’s “State-Trait Anxiety Inventory” [31], which has two parts, one to evaluate the anxiety-state, and the other the anxiety-trait. Each section includes 20 statements for which the patient has to assign a score from 0 to 3. The higher the score, the higher the anxiety. In this study, only the anxiety-state was taken into consideration. This questionnaire shows a high convergent validity with other measures of anxiety and presents high reliability with a Cronbach’s alpha of 0.94.

#### 2.7.4. Depression Level

This was measured with the Spanish version of the “Beck Depression Inventory” [32]. This inventory comprises 21 statements that are assigned a score between 0 and 3. A higher score indicates a higher level of depression: minimal (0–13), mild (14–18), moderate (19–27), and severe (28–63) [33]. The reliability values for this questionnaire are high (Cronbach’s alpha = 0.83) as well as the validity (correlations range from 0.68 to 0.89).

#### 2.7.5. Level of Catastrophizing When Faced with Pain 

This was measured with the Spanish version of the “Pain Catastrophizing Scale” [34]. It is a self-administered questionnaire that consists of 13 items (3 sections) corresponding to thoughts when faced with painful situations. The subject should respond to each using a five-point Likert scale with a response scale from 0 (none/never) to 4 (all the time). The theoretical range of the instrument is between 13 and 62, with low scores indicating little catastrophization and high values, high catastrophization. In this study, the entire questionnaire was taken into consideration. This scale has been shown to have good validity, internal consistency (Cronbach’s alpha = 0.79), test–retest reliability (intraclass correlation coefficient = 0.84), and sensitivity to change (effect size ≥ 2).

#### 2.7.6. Previous Pain History

Participants were asked if they had suffered previous events with a high intensity of physical pain (fractures, hairline fractures, dislocation, appendicitis, kidney stone, childbirth, severe back pain etc.) or any event, which they remembered as very painful. Participants were required to respond with a yes or no.

#### 2.7.7. Self-Perceived Tolerance to Pain

Participants were asked about their resistance to pain, or how well they considered that they tolerate pain. Responses were “low–medium tolerance” (believing that they can tolerate little or average pain) or “high tolerance” (can tolerate a lot of pain).

#### 2.7.8. Individual Affinity for the Stimulus

This variable aimed to determine if the individual felt a prior like or dislike of the CS. This question was only asked for the two groups that underwent the cold protocols, as ischemic nociceptive perception is a sensation that does not generally provoke an extreme appreciation of liking or disliking based on previous experience with a similar type of stimulus. Instead, the majority perceives this sensation as “indifferent” or bearable (at medium nociceptive levels), without generating significant likes or dislikes. Neither is it possible to ask about the opposite stimulus of the sensation with which it could be compared and thus make it easier to choose between like or dislike, as in the case of cold/hot water.

Participants in the water test paradigms were asked two questions: (a) if they preferred warm or cold water when getting wet or bathing and (b) which stimulus, in general, did they like more (hot or cold). If the two answers were positive for a cold stimulus, the individual was considered as having an affinity for the cold (Like), and if both responses were positive for hot, they were considered as having no affinity for the cold (Dislike). A mixed response was not considered.

### 2.8. Sample Size

The study sample size was calculated using the program GRANMO version 7 (IMIM-Institut Hospital del Mar d’Investigacions Mèdiques, Barcelona, Spain) and the estimate was based on the standard deviation (SD = 0.4) of a similar study and assuming a minimum difference of 0.47 (equivalent to 10% change) as clinically relevant for the CPM effect [35]. A required sample of 18 individuals per group was estimated. Therefore, a total sample of 72 individuals would be needed to detect significant differences in the CPM effect with 95% confidence (α = 0.05) and 80% power.

### 2.9. Statistical Analysis

Data were analyzed using the statistical package SPSS 21.00 (SPSS Inc., Chicago, IL, USA). Normality of data was tested using the Kolmogorov–Smirnov test. A descriptive analysis was conducted using means and standard deviations for the quantitative variables and frequencies and percentages for the categorical variables

To study the homogeneity of the four groups in terms of the variables at the start of the intervention, we used the chi-squared test for categorical variables and one-way ANOVA for quantitative variables.

To analyze the principal objective, a 4 × 2 mixed ANOVA model was used because the outcome variable (CPM effect) depended on two independent variables: the variable “group” (ischemic-sequential, ischemic-parallel, cold-sequential or cold-parallel), which acted as a “between” variable, and the variable “time” (prestimulus or poststimulus), which acted as an “intra” variable. The responder rate for each of the test paradigms was calculated as the percentage of all participants who have experienced pain inhibition. The effect size was also calculated using the partial eta-squared test (ηP^2^), where a value between 0.2 and 0.49 indicated a small effect size, a value between 0.5 and 0.79 a medium effect size, and a value >0.8 indicated a large effect [36].

To analyze the possible influence of confounders on the CPM effect including sociodemographic variables, healthy habit variables, anxiety and depression levels, level of catastrophizing when faced with pain, previous pain history, self-perceived tolerance to pain and individual affinity for the stimulus, a bivariate analysis was conducted using a linear regression model. The corrected or adjusted R^2^ was obtained to determine the percentage of variance that could be explained by each of the potential predictors (if several variables existed, the corresponding multivariate linear regression model would be analyzed).

The cold water test paradigms (sequential and parallel) were dealt with separately to analyze in greater depth the association of the CPM effect with the variable “individual affinity for the stimulus” (Like or Dislike). To address these possible combinations, we established four new groups that were analyzed with a new 4 × 2 mixed ANOVA model in which the “group” comprised the following variables: cold-sequential/Like, cold-sequential/Dislike, cold-parallel/Like and cold-parallel/Dislike (which acted as an “inter” variable), and the time (pre-stimulus or post-stimulus) continued as an “intra” variable. The level of significance was established at 0.05 for all tests, and the confidence interval set at 95% [36].

## 3. Results

**Figure 2:**
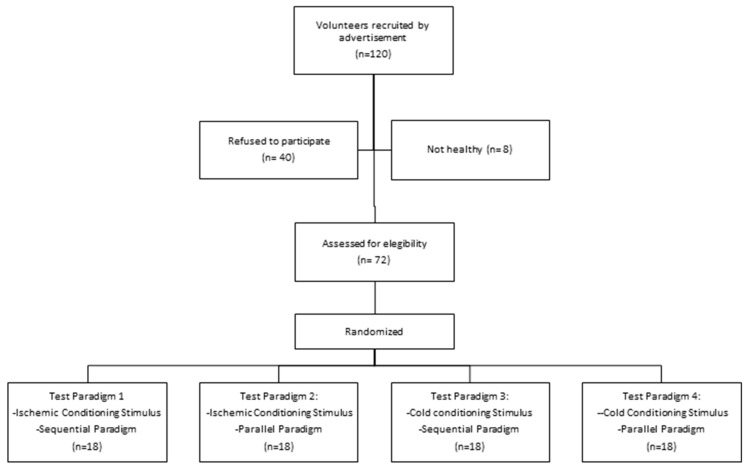
Flow-chart of the study.

A total of 120 people responded to the adverts, of which 40 refused to participate (because they were not interested in studies about pain or they had incompatible schedules). Eight did not meet the inclusion criteria. A final total of 72 healthy volunteers participated. There were no losses during the study; the study flow chart is presented in Figure 2. All participants in the ischemic groups had reached 6/10 on the NRS when they were asked (after one minute), and all participants in the cold water groups reached 6/10 on the NRS before two minutes.

All participants in the two groups with the cold water stimulus (*n* = 36) were considered in the study of the variable “individual affinity for the stimulus” as all showed either a clear affinity or no affinity for the stimulus in the two questions used to determine this.

The descriptive characteristics of the sample, overall and in each study group, are presented in Table 1. There were no significant differences between the groups at the start of the study for any of the variables (*p* > 0.05), except for the variable “individual affinity for the stimulus”, which was only considered for the cold water test paradigm, where the cold-sequential group had proportionally more participants in the category “Dislike” than the cold-parallel group (χ^2^ = 5.461, g.l = 1; *p* = 0.02). 

**Figure 3:**
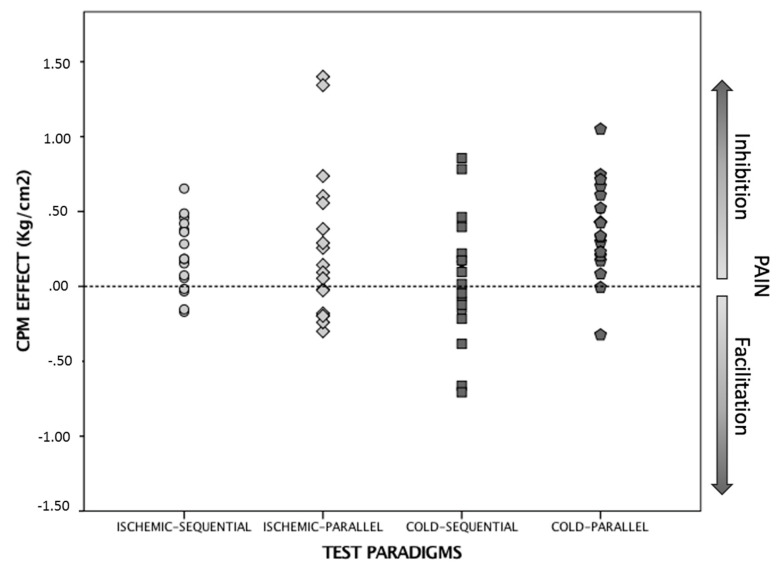
CPM effect in response to each test paradigm.

Table 2 presents the mean values and standard deviations for the variables of nociceptive pressure with the algometer before the conditioning stimulus (Pre-CS) and nociceptive pressure after or during the CS (Post-CS) for each of the test paradigms, as well as the “CPM effect”, which is the difference between these two measurements (with its corresponding 95% CI). CPM is presented as an absolute value indicating if the result denoted pain inhibition or pain facilitation (I/F). The responder rate is also expressed in the table. First, the test paradigms were analyzed for whether they produced a CPM effect and which of them was more effective in obtaining this effect. The 4 × 2 mixed ANOVA model did not indicate a statistically significant group-by-time interaction (F = 2.56, *p* = 0.06). Nevertheless, the global CPM effect showed a major time effect (F = 25.12, *p* = 0.001), which indicates that there was a change in the CPM effect in general in the study sample but without differences between test paradigms. In the four test paradigms, in global terms, the “Post-CS” mean values are higher than those for “Pre-CS”, and the CPM effect variable has a positive value, which demonstrates that the phenomenon was activated (Figure 3). The responder rate was very high for the ischemic-sequential and cold-parallel test paradigms and was moderate for ischemic-parallel. In terms of the effect size, a low value was obtained for the “ischemic-sequential” and “ischemic-parallel” test paradigms, recording values of ηP^2^ = 0.48 and ηP^2^ = 0.22, respectively, and a medium value in the “cold-parallel” paradigm of ηP^2^ = 0.61, while the “cold-sequential” test paradigm showed no effectiveness.

In the regression analysis (Table 3) studying the possible association between the CPM effect and the rest of the variables, no statistically significant association was found between the CPM effect and sociodemographic variables such as age (*p* = 0.42), BMI (*p* = 0.32), sex (*p* = 0.37), menstrual period (*p* = 0.78), hormonal contraception (*p* = 0.50), socioeconomic level (*p* = 0.70), and education level (*p* = 0.42). Neither was any association found between the CPM effect and frequency of exercise (*p* = 0.23), allergies (*p* = 0.64), smoking (*p* = 0.60), dominant side (*p* = 0.34), self-perceived tolerance to pain (*p* = 0.32), or history of previous pain (*p* = 0.09).

In our study population (healthy volunteers), no association was found between the CPM effect and level of anxiety (*p* = 0.17), level of catastrophizing (*p* = 0.14), or level of depression (*p* = 0.32), although the levels of these variables were very low in our study sample.

The regression model only found one significant association between the CPM effect and the variable “individual affinity for the stimulus”, in the subjects in cold test paradigms, with an adjusted R^2^ of around 0.45; in other words, this factor could explain 45% of the total variability of this effect. In the cold water test paradigms, when a participant did not dislike cold (preferring cold to heat), the CPM effect was increased by 45% due to the coincidence between the individual affinity for the stimulus and the test paradigm applied.

**Figure 4:**
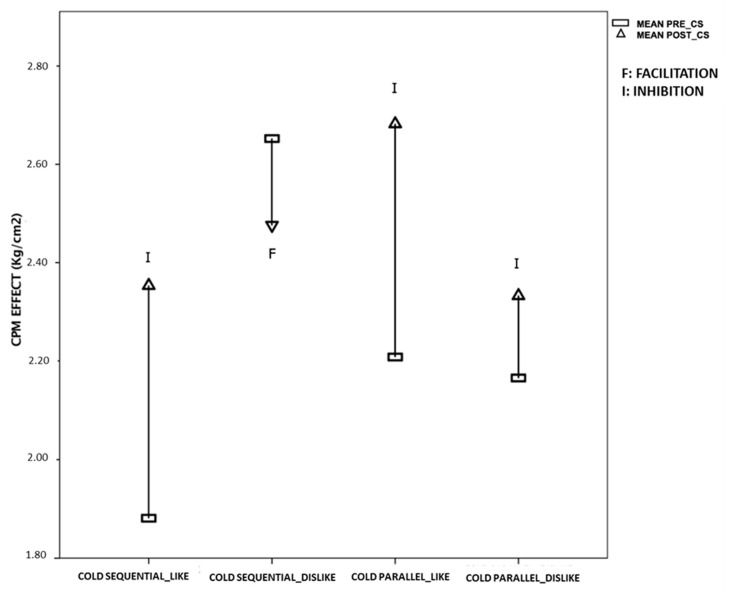
CPM effect of the cold test paradigms with the variable “individual affinity for the stimulus”.

A secondary analysis was conducted to study the CPM effect focusing on the variable “individual affinity for the stimulus” and the two cold water test paradigms, sequential and parallel (Table 4). Therefore, four new groups were made to incorporate these two factors. The 4 × 2 mixed ANOVA model indicated a statistically significant group-by-time interaction (F = 12.287, *p* = 0.001). Those participants that had a higher affinity for a cold stimulus were observed to obtain a significant and efficient CPM effect, with an effect size close to large in both the sequential-water test paradigm (ηP^2^ = 0.75) and the cold-parallel test paradigm (ηP^2^ = 0.76). In contrast, those persons that did not have an affinity for the cold did not obtain a significant CPM effect (Figure 4), independent of how the test paradigm was applied (parallel or sequential).

## 4. Discussion

The principal objective of the present study was to determine if these test paradigms produced the CPM response and to compare the effectiveness of each in terms of effect size. The secondary objective was to evaluate the possible influence of different potential predictors that may be associated with the CPM effect.

The analysis determined that there were no statistically significant between the four test paradigms in activating the CPM phenomenon (Pain inhibition). All groups showed Post-CS values higher than Pre-CS, and the response rate was greater than 60% in ischemic-sequential, ischemic-parallel, and cold-parallel paradigms. When we analyzed the effectiveness of the four test paradigms, we obtained a medium effect size in the cold-parallel test paradigm followed by the two ischemia test paradigms (small effect size) and no effect size was obtained in the cold-sequential test paradigm.

We expected to obtain the CPM effect in the four test paradigms because the study was carried out in a healthy population, so the CPM effect should be produced. Moreover, the test paradigms were based on designs from previous research, where it had already been demonstrated that pressure, cold water, and ischemic pain [10,22] were stimuli that could be used to provoke a CPM effect. Although in this study, Pain 60 was used, and the most used method is pain threshold PPT [22], it has been shown that it is also capable of inducing the CPM response. Thus, as stated in other studies: what is necessary to trigger this phenomenon is to obtain a minimum intensity of pain (and tolerable) [24]. It is hypothesized that this intensity was sufficient to induce the CPM response in our population. Similarly, it is evident that the neck on the side of the dominant arm and the foot on the contralateral side are suitable sites to study CPM, as established in previous research, in which it is assured that the location of the stimuli does not influence the CPM response, except for ipsilateral and homotopic areas [26].

A possible inconsistency that we found in the results, as it does not fit with the existing literature [6], is that the water-sequential group had a null effect. This method is widely recommended, even above the parallel method [6], since the latter may be influenced by biases such as distraction [24]. A possible explanation for these results may be because this group had a higher proportion of people who “had no affinity for the cold”.

We obtained a significant association between the variable “individual affinity for the stimulus” and the CPM effect, which indicates that this response may be stimulus-dependent, and which was able to explain up to 45% of the total effect of the following phenomenon: Those people who received the cold water CS and had more affinity for the cold had a inhibitory effect, while those who did not have an affinity for the cold had a non-significant CPM effect. An in-depth analysis of the CPM effect was performed in the cold groups (sequential and parallel) concerning the affinity for the stimulus (like or dislike). It was effectively found that people who preferred the cold (in both the sequential and parallel groups) obtained an inhibitory and significant CPM effect. However, people who did not feel this affinity for the cold did not have a significant CPM response, regardless of whether it had been carried out sequentially or in parallel. Furthermore, this seems to confirm that applying the cold CS in parallel or sequentially is not an interaction factor in the relationship between the CPM effect and stimulus affinity; it is precisely the liking or disliking that conditions the response.

Along these lines, a study by T. Ng [35] found that patients of Indian origin had less tolerance to the cold and a lower CPM effect compared to patients of Chinese or other Asian origin. Moreover, a study by J.L. Rudy et al. [37] noted that Native Americans had less tolerance to the cold than non-Hispanic whites and that this response could be because the stimulus produces a greater affective response in this group. Although both studies relate the lower tolerance to cold pain and lower CPM response with racial differences, they agree that the affective-motivational dimension of pain can contribute and that these parameters (cold tolerance and CPM response) are dependent on culture, psychosocial influence and the pain belief system.

On the other hand, the study by S. Firouzian et al. [38] measured how unpleasant CS seemed to each participant with a numerical scale (0-100). They found that greater CS pain unpleasantness was associated with lower CPM (greater pain facilitation), ‘perhaps due to the individual being in an overall negative state of arousal’.

Therefore, in our study, the lower activation of the CPM phenomenon in the cold-sequential test paradigm could be related to this pain belief system or each person’s own experience, as in this group, there was a higher proportion of people who had no affinity for the cold and who underwent, precisely, a nociceptive cold water stimulus (for them, a more unpleasant pain perception).

The authors of the present study believe that the nocebo effect may have influenced this result, that is to say, that the negative expectations of the patient regarding a treatment or procedure cause the treatment or procedure to have a more negative effect than it otherwise would have. Nocebo can be the result of expectations (people who think the stimulus is going to hurt them have higher pain scores) or experiences (people who have had negative experiences with the stimulus have more pain).

At the moment in which the patient discovers that the stimulus to which he/she is going to be subjected is not pleasant at all (in this case, cold water), the endogenous process of pain reduction may be altered. This would also indicate a possible influence of not only physiological aspects in this type of response but also cognitive aspects.

Many studies have investigated the nocebo effect and have shown how previous emotions, beliefs, or experiences influence pain-relieving responses [39]. The influence of the nocebo effect on the CPM effect has also been studied, but with a focus on how a command or negative information (e.g., “It will hurt you”, “I will give you a cream that enhances the painful sensation”) can cause the perception of pain to be greater [8,40]. No other studies have taken into account the subject’s preference regarding which stimulus is more pleasant and which is more unpleasant when it could be considered a conditioning factor for a situation in which the nocebo effect predominates.

Similarly, although the cold-parallel test paradigm had a medium effect size and could be considered the best test paradigm in our investigation for studying this phenomenon, we should take into account the finding mentioned above that there are individuals who “do not like the cold” and that this could condition the CPM effect. Although previous research has shown that that using cold water as the CS is a reliable method and produces a significant CPM effect [22,23,41], these previous studies did not analyze the preference for the stimulus and how it could directly and considerably influence the response of the phenomenon. Moreover, there is research that has reported that this test paradigm is affected by biases, such as distraction (because it is a parallel method [24]), and the fact that the cold water induces the activation of the cardiovascular system, which induces a rise in blood pressure and, at the same time, leads to an increase in the CPM effect, since both effects would be added together [9,42].

This fact can be considered to explain why the effect size in the ischemia groups was smaller than in the cold-parallel group because it has been reported that this activation of blood pressure does not occur in studies where the CS was ischemia [43]. Our findings are consistent with these results, and this suggests that the relationship between blood pressure and CPM response may be CS-dependent [43].

The secondary objective of this work was to analyze the influence of some potential predictors on the CPM effect. No significant differences were found for any of the variables evaluated except for the variable “individual affinity for the stimulus”. In terms of the age variable, we did not find differences, perhaps because, in general, our study sample was a young population, and it is known that the older the subject, the lower the CPM response [12]. As in a study by D. Locke [44], no significant differences were found in terms of gender, although there are studies that support the opposite [12]; this could be because most of the sample was male (males have a larger CPM effect) [12] and it was not a population with chronic diseases.

Although the relationship between socioeconomic status and CPM response has been little studied [12], there are studies about pain in which it is shown that lower socioeconomic levels are associated with chronic pain [45] and in our sample, most of the people had a medium socioeconomic level. Most of the participants had university or higher-level education, a characteristic that has been shown to produce a higher CPM effect [14]. It may have been for these reasons that we did not find significant differences in these variables.

The relationships between the variables smoking, allergies, physical activity and BMI and the CPM response has been little investigated [12]. In the present study, the majority of the population were non-smokers, had no allergies, had a medium–high level of physical activity, and had an adequate BMI. This is a population with healthy characteristics and therefore with a predictably efficient CPM effect. Furthermore, most of the participants had mild anxiety, catastrophizing, and depression. In the literature, it has been reported that low levels in these variables correspond to an efficient CPM response [16]. The possible influence of dominant side was studied in the study, but no statistically significant differences were found for this variable. The variables menstrual cycle (to have the menstrual period) and hormonal contraception were measured, but no significant differences were found, although there are studies that affirm that they can alter the CPM response [12,15]. Perhaps since the sample of women was very small, these differences were not detected.

It has also previously been proposed that previous pain experience and pain threshold are variables that can influence pain modulation [19,46]. Therefore, in this study, we analyzed the influence of the variables self-perceived tolerance to pain and previous history of pain. However, no relationship was found between these variables and the CPM effect. These variables have not been mentioned in other CPM studies. Although we did not find significant differences, these variables could be interesting to study the prediction of the CPM effect.

Concerning the limitations of this study, we must comment that the CPM effect was only evaluated in the short term, and there are studies that have also followed up this response in the longer term [23]. Additionally, the measure used to quantify the intensity of the nociceptive stimulus was not the pain threshold, and it was not performed at time intervals (but when they reached a 6/10 on the NRS in our case), which regularly appears in many scientific studies on this topic. The comparison of results and subsequent extrapolation for the rest of the scientific community may therefore be difficult.

As regards the strengths of this study, the participants were randomized in the distribution of the groups. Both subjects and test paradigms were identified with a random number allowing the investigator to be blinded in the analysis of the data.

Although we started with the idea that a consensus must be reached to create a single test paradigm when studying CPM, our findings also lead us to consider that this may not be entirely correct. Individual affinity for the stimulus must be taken into account to avoid drawing erroneous conclusions, such as the idea that the test paradigm used is not valid or that a given patient could have a dysfunction in the endogenous modulation pathways of pain. In this way, different, more personalized and individualized test paradigms based on the particular preferences of each patient should be used.

This factor should be considered in future research to avoid using clinical test paradigms that may not be the most appropriate for specific individuals. In the same way, this should be considered with those populations with certain chronic diseases in which it is proven that the cold adversely affects them (fibromyalgia, arthritis, etc.) [47,48]. If we applied a cold stimulus as the CS, we might obtain less effective responses for the CPM effect than if we used any other conditioning stimulus.

Future studies with larger numbers of subjects should insist on analyzing expectations in relation to the CPM response, trying to make the variable of individual affinity for the stimulus homogeneous for the study groups. In the same way, it would be interesting to assess this affinity in more conditioning stimuli, such as heat, or even in ischemic pain with other evaluating methods. Furthermore, the different test paradigms should also be studied in the same group, to analyze the possible differences between them fuller.

## 5. Conclusions

No significant differences were found between the test paradigms, although the “cold-parallel” test paradigm showed a larger effect.

No association was found between the CPM effect and sociodemographic variables (such as age or sex) or healthy habit variables, nor anxiety, depression, catastrophizing, previous history of pain, or self-perceived pain tolerance.

The like or dislike of a conditioning stimulus when conducting the paradigms, based on previous experience with a similar type of stimulus, significantly influenced the CPM effect, indicating that this effect may be stimulus-dependent. A strong association was found between the CPM effect and the individual affinity for the cold; this result could explain 45% of the total response when “individual affinity” (“like/prefer cold to heat” in this case) coincided with the conditioning stimulus (cold water).

Future research should take into account this variable (individual affinity or not to the conditioning stimulus) to strengthen the results of this study as it constitutes a substantial finding to be considered when choosing test paradigms to use in each case. An individual adaptation to the characteristics and preferences of each person could considerably optimize the CPM effect.

## Figures and Tables

**Table 1:** Sociodemographic and anthropometric characteristics and baseline comparability of all test paradigms.

Variables	Total Cohort *n* = 72	Ischemic- Sequential (*n* = 18)	Ischemic- Parallel (*n* = 18)	Cold- Sequential (*n* = 18)	Cold- Parallel (*n* = 18)	*p*-Value
**Sex**						
*Male*	45 (62.5)	12 (66.7)	10 (55.6)	13 (72.2)	10 (55.6)	
*Female*	27(37.5)	6 (33.3)	8 (44.4)	5 (27.8)	8 (44.4)	0.66
**Age** (years)	33.2 ± 12.7	32.1 ± 11.7	37 ± 16.2	29.4 ± 6.6	34.3 ± 14.1	
	(20–65)	(22–55)	(20–65)	(21–43)	(20–57)	0.32
**BMI** (kg/m^2^)	24.5 ± 3.5	24.1 ± 3.7	24.3 ± 3.5	24.3 ± 3.3	25.2 ± 3.7	
	(17.3–34.3)	(18.2–31)	(18.4–31.9)	(17.3–32.5)	(19.7–34.3)	0.82
**STAI score**	13.2 ± 9.4	13 ± 10.5	13.4 ± 9.3	14 ± 10.6	12.4 ± 7.7	
(0–60)	(0–41)	(0–39)	(2–41)	(0–37)	(3–32)	0.97
**PCS score**	12.1 ± 8.2	10.2 ± 9.2	14.8 ± 8.6	10.9 ± 8.2	12.6 ± 6.8	
(0–52)	(0–38)	(0–38)	(1–25)	(0–35)	(2–26)	0.35
**BDI score**	6.5 ± 5	6.4 ± 5.6	7.7 ± 5.7	6.4 ± 4	5.4 ± 4.6	
(0–63)	(0–27)	(0–24)	(2–27)	(1–16)	(0–13)	0.60
**Pressure pain**	2.4 ± 1	2.5 ± 1.1	2.5 ± 1.3	2.4 ± 1.1	2.2 ± 0.7	
**Pre-CS (kg/cm^2^)**	(0.7–5.4)	(0.9–5.2)	(0.9–5.3)	(0.7–5.4)	(0.7–3.7)	0.84
**Menstrual period**						
**Not applicable**	45 (62.5)	12 (66.7)	10 (55.6)	13 (72.2)	10 (55.6)	
**Yes**	7 (9.7)	1 (5.6)	4 (22.2)	0 (0)	2 (11.1)	
**No**	20 (27.8)	5 (27.8)	4 (22.2)	5 (27.8)	6 (33.3)	0.42
**Hormonal contraception**						
**Not applicable**	45 (62.5)	12 (66.7)	10 (55.6)	13 (72.2)	10 (55.6)	
**Yes**	8 (11.1)	2 (11.1)	1 (5.6)	4 (22.2)	1 (5.6)	
**No**	19 (26.4)	4 (22.2)	7 (38.9)	1 (5.6)	7 (38.9)	0.18
**Education level**						
*Primary*	3 (4.2)	3 (16.7)	0 (0)	0 (0)	0 (0)	
*Secondary*	5 (6.9)	2 (11.1)	0 (0)	2 (11.1)	1 (5.6)	
*University*	40 (55.6)	7 (38.9)	9 (50)	12 (66.7)	12 (66.7)	
*Master’s degree*	24 (33.3)	6 (33.3)	9 (50)	4 (22.2)	5 (27.8)	0.08
**Socioeconomic level**						
*Low*	5 (6.9)	1 (5.6)	2 (11.1)	1 (5.6)	1 (5.6)	
*Medium*	67 (93.1)	17 (94.4)	16 (88.9)	17 (94.4)	17 (94.4)	0.89
**Hours of exercise per week**						
*<1 h*	8 (11.1)	1 (5.6)	1 (5.6)	2 (11.1)	4 (22.2)	
*1–3 h*	23 (31.9)	3 (16.7)	7 (38.9)	8 (44.4)	5 (27.8)	
*>3 h*	41 (56.9)	14 (77.8)	10 (55.6)	8 (44.4)	9 (50)	0.27
**Allergies**						
*Yes*	21 (29.2)	5 (27.8)	6 (33.3)	6 (33.3)	4 (77.8)	
*No*	51 (70.8)	13 (72.8)	12 (66.7)	12 (66.7)	14 (22.2)	0.86
**Smoker**						
*Yes*	15 (20.8)	3 (16.7)	5 (27.8)	2 (11.1)	5 (27.8)	
*No*	57 (79.2)	15 (83.3)	13 (72.8)	16 (88.9)	13 (72.8)	0.52
**Dominant Side**						
*Right-handed*	67 (93.1)	17 (94.4)	17 (94.4)	17 (94.4)	16 (89.9)	
*Left-handed*	5 (6.9)	1 (5.6)	1 (5.6)	1 (5.6)	2 (11.1)	0.89
**Previous history of pain**						
*Yes*	51 (70.8)	10 (55.6)	15 (83.3)	12 (66.7)	14 (77.8)	
*No*	21 (29.2)	8 (44.4)	3 (16.7)	6 (33.3)	4 (22.2)	0.27
**Self-perceived pain tolerance**						
*Low–Medium*	27 (37.5)	9 (50)	8 (44.4)	6 (33.3)	4 (22.2)	
*High*	45 (62.5)	9 (50)	10 (55.6)	12 (66.7)	14 (77.8)	0.32
**Individual affinity for the stimulus**						
(Cold Test *n* = 36)						
*Like*	19 (52.8)			6 (33.3)	13 (72.2)	
*Dislike*	17 (47.2)	-	-	12 (66.7)	5 (27.8)	0.02

Data are given as mean ± SD and range for continuous variables and frequencies (percentages) for categorical variables. BMI: Body Mass Index; STAI: State-Trait Anxiety Inventory; PCS: Pain Catastrophizing Scale; BDI: Beck Depression Inventory.

**Table 2:** Descriptive data, statistical significance, and effect size of each test paradigm.

Test Paradigm	Pre-CS	Post-CS	CPM Effect Absolute Value 95% CI (Change Scores within Test Paradigms)	F/I	Responder Rate (%)	Eta Squared	*p*-Value (Global)
**Ischemic-Sequential**	2.5 ± 1.1	2.7 ± 1.2	0.2 ± 0.2 (0.10 to 0.32)	I	77.8	0.48	Group 0.90 Time 0.001 Time × Group 0.06
**Ischemic-Parallel**	2.5 ± 1.3	2.8 ± 1.5	0.3 ± 0.5 (0.01 to 0.51)	I	61.1	0.22	
**Cold-Sequential**	2.4 ± 1.1	2.5 ± 1.1	0.1 ± 0.4 (−0.16 to 0.24)	I	50	0.01	
**Cold-Parallel**	2.2 ± 0.7	2.6 ± 0.9	0.4 ± 0.3 (0.23 to 0.54)	I	88.9	0.61	

Values are mean ± SD at Pre-CS and Post-CS and mean differences ± SD (95% confidence interval for the difference) for the CPM effect. F: Results denote Pain Facilitation. I: Results denote Pain Inhibition.

**Table 3:** Simple linear regression results for CPM predictors (*n* = 72).

Variables	B	B	95% CI	R^2^	Adjusted R^2^	*p*-Value
Sex	-	-	(−0.30 to 0.11)	-	-	0.368
Age (years)	-	-	(−0.01 to 0.01)	-	-	0.421
BMI (kg/m^2^)	-	-	(−0.01 to 0.04)	-	-	0.315
STAI score	-	-	(−0.02 to 0.01)	-	-	0.170
STAI score	-	-	(−0.02 to 0.01)	-	-	0.398
BDI score	-	-	(−0.01 to 0.04)	-	-	0.315
Menstrual period (*n* = 27)	-	-	(−0.32 to 0.43)	-	-	0.784
Hormonal contraception (*n* = 27)	-	-	(−0.24 to 0.48)	-	-	0.500
Education level	-	-	(−0.20 to 0.08)	-	-	0.419
Socioeconomic level	-	-	(−0.29 to 0.44)	-	-	0.695
Hours of exercise per week	-	-	(−0.07 to 0.30)	-	-	0.228
Allergies	-	-	(−0.16 to 0.25)	-	-	0.641
Smoker	-	-	(−0.29 to 0.17)	-	-	0.601
Dominant Side	-	-	(−0.19 to 0.54)	-	-	0.339
Previous history of pain	-	-	(−0.38 to 0.03)	-	-	0.086
Self-perceived pain tolerance	-	-	(−0.10 to 0.29)	-	-	0.316
Individual affinity for the stimulus (cold test; *n* = 36)	0.549	0.681	(0.34 to 0.76)	0.464	0.448	0.001

BMI: Body Mass Index; STAI: State-Trait Anxiety Inventory; PCS: Pain Catastrophizing Scale; BDI: Beck Depression Inventory. B: Unstandardized coefficient; b: Standardized coefficient; CI: Confidence Interval.

**Table 4:** Descriptive data, statistical significance, and effect size of the groups attending to the individual affinity for the stimulus when the conditioning stimulus (CS) was cold water.

Individual Affinity for the Stimulus	Paradigm	Pre-CS	Post-CS	CPM Effect Absolute Value 95% CI (Change Scores within Groups)	F/I	Eta Squared	*p*-Value (Global)
Like (*n* = 19)	Sequential	1.9 ± 0.7	2.4 ± 0.9	0.5 ± 0.3 (0.16 to 0.78)	I	0.75	Time 0.001 Group × time 0.001 Group 0.78
	Parallel	2.2 ± 0.7	2.7 ± 0.9	0.5 ± 0.3 (0.30 to 0.64)	I	0.76	
Dislike (*n* = 17)	Sequential	2.7 ± 1.2	2.5 ± 1.1	0.2 ± 0.3 (−0.35 to 0.05)	F	----	
	Parallel	2.1 ± 0.3	2.3 ± 0.6	0.2 ± 0.3 (−0.25 to 0.58)	I	----	

Values are mean ± SD at Pre-CS and Post-CS and mean differences ± SD (95% confidence interval for the difference) for the conditioned pain modulation (CPM) effect. F: Results denote Pain Facilitation. I: Results denote Pain Inhibition.
